# Parental Acceptance of Children’s Storytelling Robots: A Projection of the Uncanny Valley of AI

**DOI:** 10.3389/frobt.2021.579993

**Published:** 2021-05-19

**Authors:** Chaolan Lin, Selma Šabanović, Lynn Dombrowski, Andrew D. Miller, Erin Brady, Karl F. MacDorman

**Affiliations:** ^1^ Department of Cognitive Science, University of California, San Diego, CA, United States; ^2^ The Luddy School of Informatics, Computing, and Engineering, Indiana University, Bloomington, IN, United States; ^3^ The School of Informatics and Computing, Indiana University, Indianapolis, IN, United States

**Keywords:** artificial intelligence, design fiction, parent-child storytelling, social robotics, technology acceptance, uncanny valley

## Abstract

Parent–child story time is an important ritual of contemporary parenting. Recently, robots with artificial intelligence (AI) have become common. Parental acceptance of children’s storytelling robots, however, has received scant attention. To address this, we conducted a qualitative study with 18 parents using the research technique design fiction. Overall, parents held mixed, though generally positive, attitudes toward children’s storytelling robots. In their estimation, these robots would outperform screen-based technologies for children’s story time. However, the robots’ potential to adapt and to express emotion caused some parents to feel ambivalent about the robots, which might hinder their adoption. We found three predictors of parental acceptance of these robots: context of use, perceived agency, and perceived intelligence. Parents’ speculation revealed an uncanny valley of AI: a nonlinear relation between the human likeness of the artificial agent’s mind and affinity for the agent. Finally, we consider the implications of children’s storytelling robots, including how they could enhance equity in children’s access to education, and propose directions for research on their design to benefit family well-being.

## Introduction

“Once upon a time” is more than an opening line to children’s stories. For many, it is a tender phrase from their fondest childhood memories, suffused with parental love. Story time has life-long implications for both children and parents. Parent–child storytelling shapes the family’s identity (Kellas, 2013), culture (Kellas and Trees, 2013), rituals (Fiese and Winter, 2009), and cohesion (Frude and Killick, 2011). From their first words, children learn conversational skills through turn-taking, joint attention, and the facial expressions of their parents or others conversing (Casillas et al., 2016; Casillas and Frank, 2017). In brief, children’s story time is often a critical practice in parenting, enculturation, and education. To make the most of children’s story time, parents have used technologies including digital books, interactive games, and talking toys.

In recent years, robots controlled by artificial intelligence (AI) have emerged as an exciting innovation for education (Fabiane, 2012; Toh et al., 2016), entertainment (Hoffman and Ju, 2014), cognitive therapy (Dautenhahn and Werry, 2004; Chang and Sung, 2013), and healthcare (Broadbent et al., 2009; Shibata and Gerontology, 2011; De Graaf et al., 2015). During the COVID-19 pandemic, the potential for deploying robots in real life was promoted (Yang et al., 2020). As Gates had predicted, robotic devices could become ubiquitous—“a robot in every home” (Gates, 2007). Thus, it is worth considering the potential acceptance and use of robots for children’s story time in the home.

Domain experts have started discussions on topics including human preferences regarding decisions made by machines (Awad et al., 2018), trust in robots (Hancock et al., 2011), explanations of robot behaviors (De Graaf and Malle, 2019), and so on. However, to ensure robots fit their context of use, their design principles should be derived from the study of their intended social ecology (Šabanović, 2010). With notable exceptions (De Graaf and Ben Allouch, 2013), few studies have analyzed sociocultural influences affecting the acceptance of robotic technology for children’s story time. This study begins to fill this gap.

As parental beliefs and values about children’s technology inform their views on appropriate use (Eagle, 2012), our research questions were as follows: 1) To what extent do parents accept children’s storytelling robots in the home? 2) How do parents envision these robots in the future? 3) What aspects of the robots could support or hinder their acceptance among parents? To address these questions, we conducted semi-structured interviews with 18 parents of children, age 2 to 5. We used design fiction, a research technique for participants to envision the use of a fictitious technology (Blythe, 2014; Lindley and Coulton, 2015).

Our main findings were as follows: 1) Despite concerns, parents were generally willing to accept children’s storytelling robots. 2) Some parents viewed the robot as their replacement, a “parent double.” By contrast, they viewed screen-based technologies as a way to keep their children occupied when they are busy with other things. 3) Parents valued a robot’s ability to adapt and express emotion but also felt ambivalent about it, which could hinder their adoption. 4) The context of use, perceived agency, and perceived intelligence of the robot were potential predictors of parental acceptance. 5) Parents’ speculation revealed an uncanny valley of AI: a nonlinear relation between the perceived human likeness of the artificial agent’s mind and affinity for the agent. Two issues that could elicit cognitive dissonance were discussed: affordances of AI and mind perception of robots. Finally, we propose research directions for designing robots that enhance family wellness and meet the needs of parents and their children in everyday home settings.

## Background

Our study connects with four bodies of work. We examine children’s story time with parents, review the design of story-time technology, investigate the role of parents in their children’s technology use, and revisit the pros and cons of existing protocols for evaluating technology acceptance.

### Children’s Story Time With Parents

Storytelling is a common way for families to spend time together. It occurs as a form of family communication in either a discursive or unified fashion. Children are exposed to 1,000 to 2,000 words every hour from parents who talk as they go about their daily activities (Hart and Risley, 1999). Regular exposure to stories promotes language acquisition (Soundy, 1993), emergent literacy (Allison and Watson, 1994; Speaker, 2000), and intellectual development (Kim, 1999). Exposure to stories helps children acquire a first language while maturing and developing (Chomsky, 1972). Parental storytelling can promote reading readiness, positive attitudes, and achievement (Silvern, 1985). Through stories told orally, children acquire syntax and listening comprehension, which later support reading comprehension (Shanahan and Lonigan, 2013).

Story time helps children learn how to make sense of their experiences and relate to other people (Wells, 1986). Children’s language acquisition occurs in the “social context of discourse, in the miniaturized culture that governs the communicative interaction of children and adults” (Bruner, 1981). The social nature of story time can support and extend children’s social life. Stories help children develop an understanding of human behavior and the world through imagination (Benton and Fox, 1985).

According to narrative performance theory, storytelling is a way of performing family identity (Langellier and Family, 2006); family storytelling constitutes children’s particular identities through content-ordering—for example, by drawing on and distinguishing social and cultural resources, such as class, race, and culture.

Bronfenbrenner’s ecological model (1979) emphasizes the centrality of the family and especially the parents in a child’s development. The ways children experience storytelling depend heavily on parental beliefs and involvement. For instance, parents may spend less time with their children as societal values shift toward individualism (Whitehead, 1991). Moreover, the goals parents set for their children’s development can influence how they interact with them (Schneider et al., 1997).

Parent–child interactions during story time, such as turn-taking, do more to support children’s language development than mere exposure to speech (Romeo et al., 2018). Additionally, parent–child attachment enhances the quality of children’s involvement in story time (Bus et al., 1997). However, Bergin (2001) found that shared reading may not be beneficial if parents are hostile and critical of their children.

In sum, story time can play a crucial role in a child’s upbringing. The efficacy of children’s story time may differ widely because of parental attitudes and involvement, resulting in complex and unequal opportunities among children.

### Technology for Children’s Story Time

The use of artifacts during story time is not uncommon. In prehistoric times, parents may have told their children stories around campfires, employing props like stones, branches, bones, and so on. In ancient times, parents read stories from papyrus. In the 15th century, the printing press enabled the spread of books, which eventually led to a flowering of children’s books, especially in the second half of the 19th century. In the 20th century, parents would sometimes use a record, CD player, or television program during children’s story time. At the advent of the 21st century, new technologies have been reshaping children’s experience. For instance, children can access storytelling with the click of a hyperlink thanks to the personal computer and the Internet.

Researchers in academia and industry have been using various technologies to facilitate and understand children’s story time. For example, stories “read” by an iPod Shuffle were found to engage and motivate K–12 students (Boeglin-Quintana and Donovan, 2013). Using videoconferencing, researchers found ways to create children’s story time for families separated by long distances (Ballagas et al., 2010). Interactive literature on smartphones and tablets has helped children improve their reading comprehension through role-playing (Borgstrom, 2011). Researchers have used 3D virtual narratives to explore children’s understanding of stories (Porteous et al., 2017). Recently, virtual assistants like Alexa were found to engage parents with their children in story time through a voice interface (Beneteau et al., 2020).

For children’s story time, one technology stands out in the progression from artifact to agent: social robots. Robotic storytellers are also part of a historical progression. The first talking dolls date back to 1890; they were made possible by the invention of the phonograph in 1871 (Plowman, 2004). In 1959, Chatty Cathy appeared as a pull-string talking doll. In 1985, Teddy Ruxpin, introduced as “the world’s first animated talking toy,” could move its mouth and eyes while “reading” stories played on a tape deck in its back. In 2002, Cindy Smart was marketed as the first doll that could recognize 650 words in English and some foreign words.

Despite warnings that electronic toys might inhibit children’s short- and long-term development (e.g., Levin and Rosenquest, 2001), AI-enabled storytelling robots have helped children learn in various ways. For instance, robots supported children’s language acquisition as learning companions in a storytelling game (Kory-Westlund and Breazeal, 2014). Affectively personalized robots can assume the role of a tutor for children’s second language learning through storytelling (Gordon et al., 2016). In recent years, Codi, Trobo, and other storytelling robots have been marketed as providing developmental support outside of the classroom. Nevertheless, the influence of these robots requires investigation.

### Parental Mediation of Technology Use

Novel technologies challenge most parents (Bowman, 2012). By letting children form more contacts outside the home, they can make it harder to establish family norms and sanctions (Lynd and Lynd, 1929). Although most parents consider educational robots beneficial, they lack confidence in their ability to join the child–robot interaction (Lin et al., 2012). Parents’ control of technology use influences the child’s development and the parent–child relationship (Giles and Price, 2008).

The way parents view technology has always been complex. For example, parents value cell phones for letting them keep in touch with their children but also worry about their effects (Boyd, 2014). Parents typically mediate their children’s use of technology, including television, video games computers, and the Internet. They implement strategies like co-using and restrictions with filters and monitoring software (Livingstone and Helsper, 2008).

A parent’s beliefs about children’s technology use could be shaped by the parent’s age, education, employment history, geographical location (Haight et al., 1999), and childhood (Plowman, 2015). Moreover, parents’ and children’s behavioral patterns can affect each other in various ways. Regardless of their involvement in child–technology interaction, parents provide support and guidance to their children, which in turn affect children’s behavior patterns and attitudes toward technology (Lauricella et al., 2015).

Owing to the increasing complexity of the technology landscape, traditional parental mediation theories need to be revisited (Jiow et al., 2017). So far, these theories have mainly examined social and psychological media effects and information processing (Clark, 2011). A common theme in this literature is that parental mediation of children’s technology use reflects their effort to mitigate its perceived adverse effects. Therefore, assessments of social acceptance of children’s technology, especially social robots designed for the home, should not overlook parental attitudes and family dynamics.

### Technology Acceptance

Technology acceptance denotes a user’s willingness to adopt a system and that system’s social and practical acceptability (Nielsen, 1993). A practically acceptable system may not be socially acceptable. Examples of social opposition include movements to ban nuclear power and genome editing. The rationale for social opposition may reflect a complex mixture of concerns, including morals, religion, political ideologies, power, economics, physical safety, and psychological well-being (Otway and Von Winterfeldt, 1982).

Researchers have been formulating various theoretical models to assess user acceptance of technology, beginning with the technology acceptance model (TAM, Davis et al., 1989). Venkatesh and colleagues compared eight prominent models to extend TAM, empirically validating the unified theory of acceptance and use of technology (UTAUT, Venkatesh et al., 2003). Due to the increasingly complicated context of use, researchers have been revising acceptance models for recent technologies, such as multi-touch displays (Peltonen et al., 2008), gestural interfaces (Montero et al., 2010), and speech interfaces (Efthymiou and Halvey, 2016). With the development of AI technologies, the ethics of adopting novel technologies are gaining more attention (e.g., Awad et al., 2018; Malle and Scheutz, 2018).

Social acceptance of robots could predict comfort with being in contact with them regularly. To succeed a social robot must be emotionally acceptable (De Graaf and Ben Allouch, 2013). Popular technology acceptance models like TAM and UTAUT were found to be limited for social robots. A study of Polish professionals’ acceptance of a humanoid robot for children with atypical development found attitudes toward technology were only a weak predictor of intention to use (Kossewska and Kłosowska, 2020). De Graaf and colleagues’ study showed the role normative beliefs play in the acceptance of social robots in the home (De Graaf et al., 2017).

Evaluating the social acceptance of robots is challenging. First, social robots are not merely a new form of technology. They embody human values through their humanlike presentation. In Japan, for example, robots were deployed in ways that reify “traditional” values, such as the patriarchal extended family and sociopolitical conservatism (Robertson, 2007). Moreover, various factors can challenge the validity, reliability, and practical applicability of evaluation methods (Lindblom and Andreasson, 2016). For example, the high cost of manufacturing sturdy robots that can function and survive in a home setting may compel researchers to rely on laboratory studies.

## Methods

To explore parental acceptance of children’s storytelling robots, we conducted a qualitative study employing design fiction, which is a form of speculative design that opens up discussions on the use of emerging technologies and their ethical and social implications (Dunne and Raby, 2013; Hales, 2013; Malpass, 2013; Cheon and Su, 2017). This activity let parents speculate on the future of children’s robots and their expectations and concerns.

### Participants

Participants received an invitation by email, campus forum, local Reddit community, or word of mouth. Inclusion criteria were adult parent of at least one child, age 2 to 5. The study focuses on preschool-aged children because they are more likely than older children to be cared for at home and because robots designed for this age group are less studied. These criteria provided a new baseline for comparing robot acceptance because previous research focused on interactions between social robots and older children (e.g., Tazhigaliyeva et al., 2016).

Eighteen parents from a midwestern city in the United States and its environs participated in the study. For the method used, we believe the sample size of 18 parent–child dyads reached saturation because, in the last few interviews, no new themes were observed. Guest and colleagues found that for interview studies saturation usually occurs within the first twelve interviews, and basic elements for metathemes emerge as early as the sixth interview (Guest et al., 2006).

Fourteen participants (77%) were mothers.^1^ Parents ranged in age from 24 to 38 (*M* = 32, *SD* = 4) and had a range of education levels from some college to a doctoral degree. Twelve were White, two were Black or African American, one was American Indian or Alaska Native, one was Asian, and two were another race or ethnicity. Half of the participants were full-time employees, two were part-time employees, four were students, and three were unemployed. Throughout the paper, we attribute quotes to a specific participant by using *M* for mother or *F* for father followed by a number.

This study was approved by Indiana University’s Office of Research Administration (February 16, 2018, No. 1801962828). Informed consent was obtained from all participants. Study protocols complied with federal, state, and university standards, policies, and regulations.

### Robots

To help parents imagine potential robot features and to inspire them to brainstorm, we searched for commercial robots that can read or tell stories. Two robots, Luka and Trobo,^2^ served as probes for design fiction (Schulte et al., 2016). We selected these robots because they vary in form (zoomorphic vs. humanoid), voice (human vs. robotic), materials (hard vs. fluffy), degree of autonomy, and ability to express emotions. Luka interacts with users autonomously. It can speak several sentences (e.g., “I am bored”) or blink to express emotions and attract attention. Luka has touch sensors distributed on its body. A small camera is mounted in Luka’s eye area, which enables it to “read” books. Trobo, by contrast, looks like a stuffed toy though with the shape of a humanoid robot. Trobo uses Bluetooth to read e-books on its phone application. To make Trobo appear to read physical books, we used the *Wizard of Oz* technique (Green and Wei-Haas, 1985). A researcher controlled Trobo’s reading pace remotely while the participants turned the pages.

### Procedure

Parents provided demographic information on their family and their experience with robots via an online survey. Then, the researcher met with parents and their child at their home or in the lab.

Upon meeting, the researcher showed parents how to use the robots and asked them to use the robots for their child’s story time. Parents were free to choose which robot to use first and how to be involved in the two child–robot interaction sessions. For example, some parents helped their child turn the pages of the storybook, while others encouraged the child to have story time with each robot independently. Each session lasted until the story finished (about 5 min) or was terminated by the parents when their child was too antsy or inattentive.

After the sessions, parents were interviewed for 20–50 min and were audio-recorded for transcription. During the interview, the child was given toys and a drawing kit to stay occupied. Some parents also brought a tablet computer or their partner to keep their child occupied so the interview could proceed uninterrupted.

The interviews were semi-structured. Parents were asked to reflect on their motivations, routines, and the technologies they used, if any, for children’s story time. Then, we introduced the prompt for design fiction, which was a narrative of their own creation: “a robot designed to read or tell children stories for daily use in the home” (Stanley and Dillingham, 2009). Parents were asked to envision the context of use, the features of a robot they would accept, and their related thoughts (Supplementary material S1). During the interviews, we avoided bringing up any specific topics, such as privacy, security, and so on. In addition, we reminded parents that they were imagining a futuristic robotic concept rather than evaluating any particular robot, including the two they had just interacted with. If the parents had more than one child, they were asked to think only of their 2–5-year-old child throughout the study. Parents received a $25 Amazon gift card as compensation.

### Data Analysis

To code the interviews, we employed grounded theory (Glaser, 1978). The first author analyzed the interview data using open, axial, and selective coding in MAXQDA (vers. 18.0.7). The other authors evaluated the trends and validity of themes. We extracted 570 excerpts from the transcripts. Through iterative memoing and refinement of categories (Corbin and Strauss, 2008), we developed three overarching themes: Story time experiences, envisioned storytelling robots, and attitudes toward using robots. Within these themes we developed further categories (Table 1).

**TABLE 1:** Data themes and examples

Themes	Subthemes	Excerpt	Sample statement
Story time experiences	Approach	67	*We’ll sit and read on my phone if we’re out somewhere*.
	Motivation	30	*It’s important for her development, for language development, imagination, and bonding*.
	Emotion	19	*I really don’t like reading some of those books over and over and over again*.
	Content	17	*We tell stories from books. If we make up stories, it’s just characters he knows, like daniel tiger or from PBS kids*.
Envisioned storytelling robots	Robot–child interaction	108	*It looks more like having a friend, having a second person than having just toys everywhere*.
	Robot appearance	36	*Maybe a size as big as a human being can be... if it is possible*.
	Robot intelligence	13	*If the child is talking back while they’re reading, the robot should be able to interact with the child*.
	Robot-delivered content	13	*Probably, no UFO stories or anything weird*.
Attitudes toward using robots	Concern	93	*I’m afraid she would lose her interpersonal skills and knowing how to interact with humans*.
	Positive attitude	88	*If he (her child) would pay attention to the robot and sit there, then I could get something else done. That would be nice*.
	Robot-related experience	65	*… cause alexa doesn’t understand the kids all the time*.
	Using robot vs. other technology	21	*The robot is made for a kid, and it had kid’s content, whereas iPads are not just made for kids. There’s lots of other stuff*.

## Findings

This section describes parents’ experience of children’s story time, their attitudes toward storytelling robots, their context of use, their vision for robot features, and their concerns. It also relates these descriptions to the literature.

### Parent–Child Story Time

Parent–child storytelling is distinguished from other family activities by its combination of instructional value for the child’s literacy and its entertainment value for both parent and child. In F2’s words, story time was special because it was “*education and playing at the same time*.” Parents commonly started reading for their children by their eighth or ninth month, if not earlier.

#### Motivation for Parent–Child Storytelling

Parents often linked storytelling to literacy education. They reasoned that story time stimulates children’s curiosity, imagination, and creativity and builds their vocabulary, all of which benefit brain development and language acquisition. M9, a children’s librarian, stated, “*All the brain synapses and connections are made in those first three years. So, it’s really important to read to them and tell them stories and have them learn as many words as possible because their brains are like sponges; they soak up everything*.” M3, a mother of a daughter who had apraxia (i.e., motor-speech disorder), explained, “*It was hard to get her to speak because what was going on in here was processed differently. Reading out loud is very good for her because it’s teaching her lots of words*.”

Some parents avoided telling their children stories because they thought it might harm their literacy. F1 explained, “*I’m really bad at phonetics, because I’m really bad about adding letters. So, I’d feel bad if he walked into school and said whatever word it was, and people made fun of him. That would stink.*” This indicates how some parents tried to balance being a help or hindrance to their child’s development of literacy.

Parent–child story time was often valued as a family tradition and time for bonding. M8 stated, “*My dad did it for me, and so it kind of reminds me of that time when I was a kid and my dad was lying in bed with me reading to me. Like a cultural tradition*.” F3 explained, “*I grew up on them. It’s kind of a staple of growing up, having stories read to you by your parents.*” Story time builds closeness and is one of the joys of parenthood. M11 said, “*We cuddle, so we sit close together, and I’ll put my arm around her, and it’s just kind of a bonding time.*” Some parents simply enjoyed story time. F1 stated, “*It’s fun. We kind of lie in bed together and look at the pages and talk about it.*” In other words, watching children get excited about stories and learn things was a treasured part of parenthood:

*I like watching the look on her face, ‘cause sometimes she’s confused, and I see her eyebrows go up and down and see her cock her head to the side. I just enjoy seeing her reaction and how curious she is. She’s curious, cocks her head, moves her eyebrows. It’s like I can see the wheels turnin’. I don’t know what they’re doing up there, but she’s definitely thinking.* [M14]

Some parents use story time to set up their child’s evening routine. As part of a bedtime ritual, parents use storytelling to help the child regulate affect (e.g., to settle down). M3 stated, “*When it’s story time, she’s not up running around and playing. And she knows that story time is the progression in going to bed.*” This ritual could also give children a sense of being part of a family and help to maintain the parent–child bond (Franklin and Bankston, 1999). Other parents hoped that their children would form healthy long-term habits through an early attachment to books. M14 explained, “*We want her to be curious and learn, and we know that it starts at a young age. So, by reading stories to her now, we hope she’s going to continue to want to read and continue to want to learn.*” These examples reveal storytelling as a way of parenting.

Stories took various forms. Some parents read fairytales or short stories because they fit their children’s attention spans. Other parents told family stories. Their children enjoyed hearing about what happened before they were born. In particular, parents tended to involve younger children in the storytelling process, such as setting a scene, developing characters, creating a plot, and so on. In other words, they created stories with their children, instead of for them. This made story time a venue for self-expression as well as social interaction, which situated the children as active agents in constructing their sense of self (Korn, 1998):

*Sometimes, she’ll say, ‘Mom, tell me a story,’ and I’ll say, ‘Okay, once upon a time there was a princess named Charlie [her daughter’s name],’ and then I’ll say, ‘And then what happened?’ and then she’ll say, ‘Oh a big dragon came and took her away,’ and so we just kind of create stories doing that. As she got a little older, we would read books more* [M11].

In sum, parent–child storytelling could serve multiple goals, involve dynamic interactions, and nurture mutual well-being. Balancing its contribution to a child’s literacy with potential adverse effects could pose a challenge to some parents.

#### The Role of Technology in Story Time

Most parents preferred the use of physical books to technology. M4 stated that, although using physical books is “*old-fashioned,*” it is something children “*have to get used to, though things are becoming more and more digitized.*” A recurring reason screen-based devices were not favored was that parents doubted young children could benefit from stories played on such a passive medium. M14 explained that “*at her age, she’s having fun [playing with a smart device], but she’s not learning anything.*” She emphasized that during story time children should physically “*experience things*” like turning pages. Similarly, F1 observed that his son didn’t look at the pictures in the book while listening to the story on a phone application. In this case, F1 mentioned, “*I try to make him pause and look at the scene that’s on the page and kind of get an idea of reading comprehension, I guess, so that these words go with this picture.*” Indeed, brain connectivity in children increased with time spend reading books and decreased with time spend using screen-based media (Horowitz-Kraus and Hutton, 2018).

Nevertheless, some parents valued smartphones and tablet PCs such as Kindles and iPads for providing quick access to a large supply of reading material. M8 stated, “*We do actually read on my phone a lot. We’ve got one of those Kids Zone type apps, and it’s got different books in there. We’ll sit and read on my phone if we’re out somewhere. It’s just easier than dragging three or four books around.*” Some parents used multimedia, such as online videos and TV programs, as a replacement for story time to relieve the stress of parenting. M9 explained, *“The screens are the only thing that can take his attention to the point where he won’t keep asking me questions. Because he’s an only child, it’s just me and him in the house. And he wants to interact with someone*.” M5, an exhausted mother, said, “*I don’t have time to tell him a story. Those programs are already on the TV. Basically, I would rather have him once in a while sit down and listen to stories*.” These accounts reflect how parents employed technology in “digital parenting” (Mascheroni et al., 2018).

### Positive Attitudes Toward Children’s Storytelling Robots

This section reports parents’ vision of children’s storytelling robots and the robot’s context of use. Parents generally held positive attitudes toward the robots, especially compared with screen-based technologies.

Parents’ positive attitudes were exhibited by their vision of the robot’s role and suitability for children. Parents envisioned a storytelling robot as a “parent double.” For instance, M4 stated, “*If I had an especially busy night, and my husband wasn’t home, and it was time to do story time. You know, that would maybe give me 10 min to pack their lunches while they listen to their story from the robot.*” Moreover, parents reported a desire to have a storytelling robot deal with “*boring tasks*” like rereading a book. M12 stated, “*If she gets to a point where she does want to do the same book, you know. Finish it and start right back over, a robot would definitely do that. I would not want to*.” Indeed, developmental studies suggested that requesting repetition in book reading is common for young children (Sulzby, 1985). Having the same book read to children repeatedly can increase their enjoyment and helps them learn new words (Horst, 2013). M10 also expected a robot to do something she didn’t enjoy doing during story time: “*My older one is almost doing chapter books. It would be awesome if [the robot] read chapter books because that’s what I hate—reading out loud*.” These examples show how parents value a robot contributing to children’s story time and reducing their stress.

Some parents wished to delegate tasks involving emotional support. M4 explained that when children need to study, “*it would be nice to have an automated thing that could do that with them, and say, ‘Hey, that’s right!’ or ‘No, that’s wrong.’ You do the boring stuff, robot, and I do the fun stuff*.” Surprisingly, some parents wanted the robots to cover difficult topics like sex and death. M3 remarked, “*She was just watching Daniel Tiger, and they were talking about death, so [the robot] could cover topics that are hard to discuss [or] at least start the conversation.*” These findings show the need for robots to perform social support.

Parents thought the robot could facilitate children’s story time in many ways, most of which relate to their perception of a robot as social. M13 explained, “*[The robot*] *would probably keep [my son’s] interest a bit longer. He might think,* ‘*It’s a person. I have to stay here because it’s reading to me.’ He might be more fascinated with it too.*” Parents speculated that robots would engage children in social interaction while reading: “*an advantage is that your child is hearing somebody else talk*.” Parents envisioned robots acting as an educational peer. M3 observed, “*I could see that being useful when they’re learning to read just because it would be a buddy to practice with, and it could maybe help her if she got stuck on a word.*” Similarly, M4 looked forward to robots that could reinforce her children’s foreign language learning at home. F1, who was not confident with phonetics, said, “*If I’m stuck on a word going, ‘I’m not sure buddy,’ then we could be with the robot and put him in front of it and he could read the paragraph. That would be really nice.*” Some parents suggested that robots could be an authoritative social mediator:

*Sometimes, when I’m reading with my five-year-old, she doesn’t believe that a word is pronounced the way that I’m pronouncing it, and so I have gone on* *dictionary.com* *and had to play it for her. And, I’m like, ‘See? Right here.’ And, she’s like, ‘No, no, no. You’re just doing that.’ So, I’m kind of like, ‘Well, see the robot said this is how you pronounce the word.’ And, she might be like, ‘Oh,* okay.’ [M10]

Parents indicated that robots would be more child-friendly than televisions, tablets, smartphones, and other devices because a robot’s predetermined content was controllable and trustworthy. M4 explained, “*I would kind of trust a pre-programmed thing made for kids, whereas something like YouTube, they could go down a wrong path and see things that they shouldn’t be seeing*.” As such, parents preferred a robot that, unlike today’s Internet (Nikken and Jansz, 2014), would not require their direct supervision. Some parents envisioned interacting with a robot would benefit children’s development by reducing their screen time, which is “*bad for their eyes*” (M10). Moreover, robots were seen as addressing usability issues that impede children’s technology adoption. M13 mentioned, “*He gets upset because he doesn’t understand how to control the phone. A robot would be easier for kids to interact with.*” This example shows how parents envisioned it being immediately apparent how to use the robots. In other words, they expected them to have highly salient affordances.

### Children’s Storytelling Robots: Expectations and Concerns

Despite the perceived usefulness of children’s storytelling robots, we observed a series of technical and social challenges that would affect parental acceptance and adoption. Previous studies indicate that people expect robots to have social traits that help them empathize with people (Breazeal, 2004). The present study found that parents expect storytelling robots to have social intelligence. However, a mechanical robot possessing this human quality might give parents cognitive dissonance. This psychological pain arises from inconsistent cognitions (Festinger, 1957), such as perceiving the robot as a social being while knowing it is just a machine. We identified two key factors impacting cognitive dissonance: a robot’s 1) adaptive capability and its 2) affective capability. Parents expected a storytelling robot to be competent at both, though their acceptance of storytelling robots could be hindered by ethical concerns and by the uncanniness of robots that seem to be electromechanical yet possess conscious experience.

#### Adaptive Capability

We define *adaptive capability* as the ability to adapt autonomously to a real-world context. Parents doubted whether the adaptive capability of robots could meet the challenges of children’s storytelling. A major concern was impromptu conversations between the child and the robot. In particular, parents expected robots to respond to questions from children automatically. Enhancing the robot’s autonomy would likely increase its perceived usefulness and thus acceptance (Thrun, 2004).

Prior research indicates that conversational interactions during story time lead to children’s literacy success (Berk, 2009). These interactions happen naturally during parent–child storytelling. For example, as M6 explained, “*When you are reading to children, they want to talk. They will not just sit and not talk as you’re reading. Most times, they want to talk like ‘Oh am I flying? Am I ...?*’” Thus, she envisioned the robot interacting in real time to help children engage with the story. “[*Children*] *usually have questions when they are reading, and if the robot is not answering the questions, they can’t even think about their questions and all*.” Discussions could help children interpret the story, including the character’s facial expressions. M13 suggested, “*The discussion is definitely important because she needs to be able to look at somebody and know if they’re angry or if they’re sad*.” Parents talking to their children would help them develop literacy and a love of reading (Burns et al., 1999).

Additionally, parents mentioned that a robot would need to recognize a toddler’s voice. M4 said, “*We have an Echo up there, and so, a lot of times when we ask Alexa questions, she’s like, ‘Oh, I don’t understand you. I don’t understand you,’ especially with the kids.*” She further explained that a child’s voice is “*so different and high-pitched, or they don’t pronounce words right, so I could see that being really frustrating for a kid if the robot’s not understanding them.*” The robot would need a system for understanding a child’s speech.

Another challenge was whether storytelling robots could maintain children’s engagement to guide their attention. In real life, parents often use linguistic skills, emotional expression, and gestures to increase engagement. For example, M5 added rhyme so that the story is “*not going to be so boring*” and to keep her child on track with the story*.* She insisted that the skill of storytelling is unique to “*human beings*.” Some parents upheld that humans were naturally better storytellers than robots because they can gauge the child’s reactions and decide what information is important to convey. Another common concern was that the robot’s synthesized voice with its flat intonation and monotonous rhythm would hamper reading comprehension. Indeed, a storytelling robot’s intonation and emotion predict concentration and engagement (Kory-Westlund et al., 2017).

Paradoxically, some parents preferred a robot with a low level of autonomy to converse with their child on security and ethical grounds. Parents feared that children trusting the robot could put them at risk if someone hacked the robot or recorded their conversations. F3 noted, “*If the robot was like, ‘What’s your dad’s social security number?’ I might freak out*.” F2 pointed out that some questions (e.g., sexual orientation) were too sensitive for young children to discuss, even with parents. M7 thought a robot with a high level of autonomy would be threatening: “*I don’t want something that’s going to take over my house. I don’t want her to become reliant on a robot.*” Given these concerns, parents suggested that stories requiring limited turn-taking would befit robot–child storytelling. Specifically, alphabet books and nursery rhymes are good candidates because they are easy to follow without a back-and-forth conversation.

A few parents found the idea of a robot talking with a human spooky. M9 remarked, “*It’d be like ‘Hello Emily, how are you?’ And I’d be like ‘No! Stop talking to me.’ But it’s just because I watched too many scary movies when I was a kid, where things that weren’t supposed to talk started talking*.” Some parents recounted their impressions of early talking toys:

*You remember those Furbies? Those things would just start talking out of nowhere, and it scared people. If the robot could just wake up and start telling a story in the middle of the night, if it started talking on its own out of nowhere, I think I would be scared of it.* [M8]

Some parents wondered how storytelling robots could flexibly adapt to complex surroundings. M14 noted that a typical story time for children would be at bedtime in dim light. The robot may be unable to “see” the book. Or, if a robot could only read in the sunroom, it could cause frustration because the sunroom might be occupied. The children might have to choose between being with the robot or being with their parents, and “*sometimes they just want to be close to mommy and daddy. They don’t want to be left alone*” [M14]. In other words, the adaptive capability of a robot could affect its actual use and even make humans accommodate to it.

#### Affective Capability

We define *affective capability* as the robot’s ability to express emotion appropriately, to influence the user’s emotional engagement with the robot and topic or story, and to arouse the user’s affection. To these ends, parents expected the robot to employ various features, such as appearance, emotion contagion, empathy, and social engagement.

Parents generally expected the appearance of a storytelling robot to be anthropomorphic, for example, “*having head and body, such as R2D2 in Star Wars*” [F3]. Some parents thought having arms and legs could differentiate a robot from other devices. In particular, robots with eyes were believed to help children take their reading time seriously. M5 reasoned, “*I like the fact that the robot has got eyes, because it looks like, ‘Okay, we are looking at each other, so what are we talking about?’ And then you could say, ‘No, that’s not a toy.*’” M11 expected the robot to have hands to hold up a physical book and turn pages so children “*have to stay engaged*.” Parents tended to imagine the robot resembling a human to support social engagement.

However, some parents felt threatened by the idea of the robot having a humanlike appearance because the child might treat it as an alternative parent. M11 was concerned that a child would think a humanoid robot was the one “*to get reading from or to spend time with.*” M13, who thought of reading as mothering her child, explained that if the robot were not humanlike, she wouldn’t feel it was taking her spot. Furthermore, several parents pointed out that robots would be creepy if they were too lifelike. One mentioned Teddy Ruxpin, an animatronic toy in the form of a talking bear: “*It’s just trying too hard to be human. I would like the toy to acknowledge that it’s a toy. I would want it to have some kind of a toy appearance*” [M12]. In particular, a parent expressed her eerie feeling when Teddy Ruxpin rolls its eyes and moves its mouth while talking:

*They were scary. I never wanted to own one, because it was just like these big eyes that moved around and this tiny mouth. It was like ‘Hi, I’m Teddy Ruxpin.’ And I was like, no you’re possessed by the Devil. *[M9]

Parents expected storytelling robots to act expressively. They emphasized that the manner of expression was critical. In M5’s words, “*Robots should be able to express stories while they are saying them.*” This aligns with Bauman’s “performance-centered conception of verbal art,” which holds that “the formal manipulation of linguistic features is secondary” (Bauman, 1977). Specifically, parents envisioned storytelling robots as being able to express emotions through body language, gestures, eye gaze, and so on. Such communication would help make concepts easily understood by young children. Children should not be “*just like sitting, watching something on a screen, or just like listening to a tape play*” [M9]. A typical example would be indicating with the hands an object’s size or shape.

Moreover, parents anticipated that emotions serve shared psychological and physiological functions, which are critical in the context of storytelling. For example, humor can hook children into story time, enhance learning, spark social interactions, and establish rapport (Savage et al., 2017). However, some parents indicated that, because humans and robots lack a shared social grounding, they would not be able to share in each other’s emotions. F3 reasoned that humor could give different people different perceptions. He provided an example where a robot was reading a picture book where characters were waving long arms: “*He can’t necessarily ask the robot why the arms are so long and squiggly or laugh with it that the arms look squiggly and funny. That’s something that humans would be like, from one person to another, subjective, but long, squiggly arms are funny. So, you can’t really have that emotional interaction with the robot.”* In sum, an emotional exchange requires social grounding, which remains a nearly insurmountable challenge for social robotics.

The affective capability of the robot led to another struggle concerning the relationship between robot and child. Some parents wanted the robot to have the affective capability to engage children in social interaction beyond story time. Unlike another device that might just be left on a shelf, they proposed children could carry the robot around, talk with it, and cuddle with it while going to sleep. However, parents worried whether interacting with the robot would impede the development of social skills or cause social dysfunction. M7 was reluctant to use the robot “*because I’m afraid she would lose interpersonal skills and knowing how to interact with humans.*” M11 highlighted human–human interactions: “*that cuddling, that hug, I think that’s important when they’re young.*” Parents commonly noted that, for young children to learn social cues and how to engage with others, real people were irreplaceable.

The idea of social robots that simulated human warmth perturbed some parents. In particular, some busy parents worried that leaving children to such robots might create a gulf between parent and child. The child might prefer the robot to the parents and spend more time hugging and cuddling with the robot. In this case, young children could switch their attachment from their parents to their robot. M4 worried that if she left her son with a robot, “*he might just get addicted to it and want to spend more time. He might find the robot’s stories more interesting than your stories, even when you have the time to tell him stories.*” M5 worried that using a robot could reduce time spent with children and chances to get to know them: “*So, they may have imaginations that you cannot know because you are not the one telling them the story.*” These parents viewed affectively capable robots as a threat to parenthood.

Some parents disliked the idea of children’s storytelling robots. They considered story time a unique part of parenting that should never be handed over to an AI-enabled robot. F1 explained, “*I think [story time] is my bonding time, this is my time to spend with my kid. So, I don’t want people to use them to separate themselves, because I feel that raising a kid is very personal.*” M9 insisted that she would never give up any story time with her child, “*I love telling stories to him, and I love reading books to him. It is my job at home*.” F4 discussed stories as a way to shape his children’s morality, stating, “*I don’t want to get to the point where robots are informing my child on topics. It’s a parents’ job to parent and be responsible for their kid. I don’t ever want that responsibility to be on a school or a robot or anything but my wife and me.*” These quotes underscore how some parents consider parent–child story time to be an activity exclusively for family members.

## Discussion

In this section, we discuss parental acceptance of children’s storytelling robots in the home. We argue that parents’ expectations and concerns reflect an uncanny valley of AI, which may be interpreted using the metaphor of the *castle in the air*. Finally, we explore the implications of designing children’s storytelling robots and propose directions for future research.

### Parental Acceptance of Children’s Storytelling Robots

Our findings indicate that, despite reservations, parents would generally accept storytelling robots in their home. Their acceptance relates to how they valued children’s story time. Parents emphasized their three main goals: literacy education, family bonding, and habit cultivation. Correspondingly, parents valued storytelling robots for pedagogy, felt they could threaten parenthood, and struggled with their potential effect on child development through daily use.

Parents also viewed parent–child story time as personally fulfilling and beneficial to their family. This explains the reluctance of some parents (e.g., M9, F1, and F4) to use a storytelling robot: It might steal from family time and weaken family cohesion. This concern is not without merit. Previous studies indicate task persistence reinforces attachment (Bergin, 2001). In other words, family cohesion increases with the time parents spend telling stories to their children. Family bonding during parent–child story time includes talking, cuddling, and joint attention, all occurring in a physically and socially shared space. Beyond giving comfort, parent–child touch could enhance prosocial behavior. To replicate this between child and robot is challenging (Willemse et al., 2017). In addition, storytelling gives parents a chance to start discussions that teach their children values. Thus, it supports family cohesion and shapes family identity.

According to parents’ narratives, a storytelling robot may require intentional agency, which assumes a strong AI position (Searle, 1980). The robot might then be able to establish an intricate microsocial environment for human children in the home. Although some parents viewed this conception as utopian (Segal, 1986), the context of children’s storytelling touched a nerve. The theory of *Ba* proposes that a living system maintains self-consistency by the contingent convergence of the separated self and the non-separated self (Robertson, 2007). Here, futuristic child–robot storytelling is a *Ba* that involves a dynamic tension between a roughly human storyteller and a developing human child. Some parents seemed disturbed by the thought of a robot guiding a child through emergent, uncertain states of development. Their concern runs counter to the expectation that a storytelling robot serve as parent double. These conflicting cognitions elicit the psychological discomfort of cognitive dissonance. Storytelling as the context of use could be a critical factor in parents’ ambivalence regarding robots for children.

We interpreted a parent’s expectation as reflecting different perspectives on a robot’s ontological status—whether it could exhibit human likeness, agency, and emotions. In the storytelling context, parents spontaneously imagined the robot as anthropomorphic and anthropopathic. Parents tended to imagine child–robot interactions as mirroring human–human interactions such as turning the pages of a physical book. By contrast, screen-based technology typically involves a graphical or voice interface. These interfaces are often not child-friendly, which frustrates parents (e.g., McFarlin et al., 2007). Thus, a robot was deemed more suitable. Parents went on to envision scenarios where the robot acts as a peer or mediator. Parents thought a physical robot could engage socially with children and forge a relationship with them, which could benefit children’s overall development. Thus, parents’ perception of robot agency heightened their expectations of the future of child–robot interaction relative to other technologies.

However, some parents were also disturbed by the scenario of a robot simulating human interactions. Nevertheless, parents seemed optimistic about the ability of futuristic robots to reduce their parenting stress by serving as a “parent double” in performing “boring” or “difficult” tasks. They expected a robotic storyteller to simulate a human storyteller’s physical autonomy and social intelligence. Successful storytelling would involve verbal and nonverbal interactions laden with affect. In other words, to be effective, a storytelling robot needs to respond dynamically to young children, whose communication involves various resources, such as gestures, vocalizations, facial expressions, body movements, and so on (Flewitt, 2006). However, parents’ ambivalent, paradoxical feelings may have been sharpened by the robot’s perceived intelligence. They struggled with its adaptive and affective capability. Parents worried children would trust the robot and follow its instruction, which could be a security threat if, for example, another person took control of it. Thus, perceived intelligence could be a third influential predictor of parents’ acceptance of storytelling robots.

In sum, the context of use, perceived agency, and perceived intelligence of a robot were promising predictors of parental acceptance. Designers of children’s storytelling robots should consider these factors in the design and evaluation process.

### A Projection of the Uncanny Valley of Artificial Intelligence

We argue that the two factors impacting parents’ cognitive dissonance, a robot’s adaptive capability and its affective capability, are a projection of the uncanny valley of AI. Why did some parents envision a children’s robot telling stories in a humanlike way—flipping storybook pages, pointing out illustrations, acting out scenes, and responding to disinterest or spontaneous questions—yet preferred the robot to have a low level of autonomy? Why did some parents feel weird when greeted by a robot but not when it told their children stories?

Consider the Chinese idiom *castle in the air*. It denotes the impractical dream of building a magnificent third floor before the first two floors are complete. Imagine building a three-story castle of a robot’s intelligence. The ground floor is weak AI. The robot combines bottom-up processes, each designed for a particular task. For example, a robot obeying the command *read a story out loud* or *open the window* might just be simulating some disconnected aspects of human behavior. The next floor is strong AI. The robot has—or at least simulates—general human intelligence. It can apply top-down processing to figure out what to do in new situations. For example, the storytelling robot may be able to infer disengagement when the child responds slowly in a low voice. The top floor is social intelligence. The robot creates the feeling of being in the presence of a living soul—with free will or whatever being human entails. For example, if the child lost interest in a story, the robot would be able to respond like a real person. Science fiction has dramatized the top floor. In the 2001 film *AI. Artificial Intelligence*, David, the robotic boy*,* felt desperate about his human mother abandoning him and set out to find out why.

**FIGURE 1:**
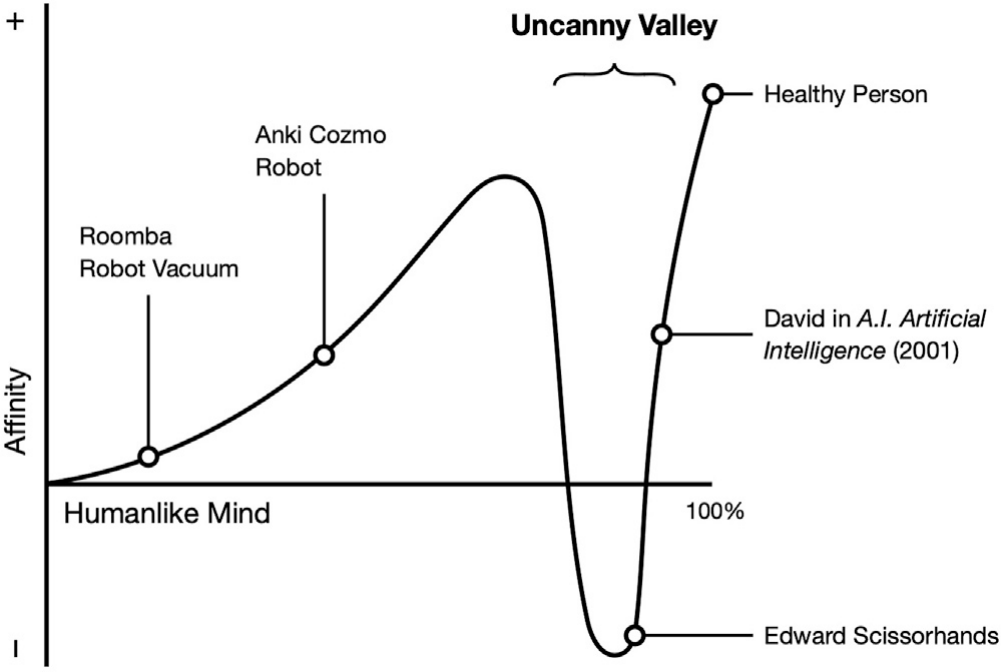
The Uncanny Valley of AI.

As the floors of the castle of a robot’s intelligence are constructed, and as its human characteristics increase, human perception begins to apply a model of a human other to the robot. If the bottom or middle floor is perceived as incomplete, the top floor becomes a castle in the air. It is unconvincing and even creepy. These perceptions reflect an uncanny valley of AI: a nonlinear relation between affinity and the perceived humanness of an artificial agent’s mind (Figure 1).

Our proposal is a bit different from Mori’s original concept of the uncanny valley of human likeness—that is, outward resemblance (Mori, 2012). Specifically, as the intelligence of an artificial agent increases, it becomes more likable, up to the point at which its perceived intelligence begins to approach human intelligence. For example, an embodied storytelling robot capable of conversation while flipping pages, is more appealing than Roomba, the cleaning robot, because the former is capable of social interaction. However, when a robot’s intelligence seems more human, but is still distinguishable from human, it could elicit an eerie feeling. This could explain why some parents preferred a robot with a low level of autonomy. For a more extreme example, Edward Scissorhands’ human intelligence is betrayed by his atypical way of thinking and behaving, which makes audiences see him as uncomfortably deviant (Clarke, 2008). This uneasy feeling disappears when a robot’s perceived intelligence becomes indistinguishable from a real human (e.g., when an AI passes the total Turning test, Harnad, 1991; Saygin et al., 2000; Turing, 1950).

Researchers have proposed different explanations of the uncanny valley (for a review, see Kätsyri et al., 2015; Wang et al., 2015). Most theories focus on how imperfect human appearance or movement triggers eeriness (Ishiguro and Dalla Libera, 2018; Paetzel et al., 2020). For example, when a robot’s human resemblance exceeds a certain point, the expectation of human performance eclipses the robot’s ability to perform (MacDorman and Ishiguro, 2006). Indeed, the idea of androids alarmed some parents (e.g., M9 and M12).

However, just as a real human being can be evaluated from the standpoint of mind or awareness, a robot’s intelligence could play a role distinct from its appearance. People might be unsettled by AI-enabled voice assistants like Amazon’s Alexa, although the shape of the device is just a black cylinder (Thakur, 2016). Factors other than a humanlike appearance influence mind perception (Gray et al., 2007; Gray et al., 2012). To extend Mori’s observation, the uncanny valley of AI predicts that 1) a certain level of intelligence facilitates social interaction between humans and robots and 2) artificial intelligence that is similar to, but still distinguishable from, human intelligence could create the uncanniness of a castle in the air.

Perceptual issues with the first two floors of a robot’s intelligence could lead to this. For example, why did a parent (M8) mention that it was creepy for Furbies to suddenly start talking? There could be at least two reasons. One is the lack of transparency of the first two floors (Kory-Westlund et al., 2016; Wallkötter et al., 2020). The affordances for engaging with the robot’s intelligence are unclear. For example, ordinary people have a limited understanding of the structure of the second floor (i.e., the robot’s perceived capability for top-down processing), which makes it difficult for them to establish a mental model of how a robot with a social capability operates (i.e., the top floor). As such, robots with the appearance of social intelligence could create an illusion. When our brain tries to falsify the illusion but fails, our expectations falter, our brain’s prediction errors accumulate, and our feeling of a social connection with that robot oscillates between what Quinton (1955) called perceptual presence and pure thought. One practical way to relieve a user’s weird feelings about a robot with high intelligence should be to make its AI understandable (Wang et al., 2019).

The other issue underlying the creepy feeling of a robot that suddenly starts talking could relate to mind perception, namely, the eerie feeling caused by the attribution of mind to a machine (Gray and Wegner, 2012; Appel et al., 2020). People may perceive mind along two dimensions: *experience*, the capacity to feel and sense, and *agency*, the capacity to act and do (Gray et al., 2007). In our findings, parents’ linguistic use reflects these two dimensions of mind: adaptive capability relates to agency and affective capability relates to experience. A robot with weak AI is perceived as being low in experience but high in agency. The increasing perception of a robot’s experience and agency tend to reinforce each other, creating a halo effect (Nisbett and Wilson, 1977).

For example, when a storytelling robot starts talking spontaneously, perhaps merely due to a bug in its program, it can create an illusory experience: The robot appears to be more than it is (i.e., the ground floor where a robot tells stories). Contradictory perceptions and cognitions cause cognitive dissonance. The robot seems to have the capacity to act and to do something unknown (i.e., the top floor). Parents are especially unsettled by unpredictable actions as they relate to their children. Because the middle floor of the robot’s intelligence (i.e., strong AI) does not yet exist, people construct the top floor as a castle in the air, which is unnerving. However, more empirical research is needed to examine the interaction between a robot’s perceived experience and agency.

### Children’s Storytelling Robots: Implications and Future Directions

Although the development of robots as a parent double still faces technical and design challenges, their social and economic value is clear. Not every child has a caregiver with leisure time for storytelling. Across the globe, we find social crises involving children: children in orphanages and other institutions—and sometimes refugee camps—without parental love and nurturance; children in foster care, perhaps separated from abusive or neglectful parents; children whose parents are illiterate, blind, deaf, or mute; children with autism who find it easier to interact with a robot, and so on (Scassellati et al., 2012). While artificial love may never replace human love, storytelling robots could lessen inequality by simulating parental warmth during early development.

The prospect of leading educational activities in the home causes some parents stress (Deniz Can and Ginsburg-Block, 2016). Alternatives, such as having relatives or babysitters read to their children or placing their children in literacy programs, may raise issues of trust or pose a financial burden. Sometimes a human assistant may be unavailable, such as during the lockdown period of a pandemic. Thus, robots acting as a parental double during story time could help relieve parental stress.

Using storytelling robots to address the social crises mentioned above is not whimsical. Robots have been used to address social crises elsewhere. For example, Japan’s government identified robotics as a solution to its looming demographic crisis caused by a lack of young people to care for older adults. Robots are also used in Japan to care for children, to provide companionship, and to perform chores (Robertson, 2007). Robots were preferred as home healthcare workers to Asian foreigners. They were considered less likely to violate cultural norms or interpret history in a way that could cause conflict (Robertson, 2007). However, social acceptance of robots could vary with religious and cultural history, personal and human identity, economic structure, professional specialization, and government policy (MacDorman et al., 2009). Thus, crosscultural issues should inform future studies on the acceptance of children’s storytelling robots.

Nevertheless, envisioning a storytelling robot has raised concerns (e.g., for M9, F1, F4). For example, a robot could become a threat to parenthood or parental identity if a child shifted attachment to the robot. A young child could develop a closer relationship with the robot through even boring activities like reading a book repeatedly (Sharkey and Sharkey, 2010; Kory-Westlund et al., 2018). The embodiment and voice of a robot could be powerful indicators of social presence (Reeves and Nass, 1996). Therefore, interacting with a robot during story time could give a young child the illusion of rapport (Turkle, 2007). However, child–robot rapport is unlikely to threaten child–parent attachment. Children are predisposed to be attached to their mother, attachment has survival value (Bowlby, 1977), and begins *in utero* (Sullivan et al., 2011).

Future directions for designing children’s storytelling robots include research on how to create educational and affective experiences for at-risk young children, how to promote the well-being and quality of life of parents, and design principles for healthy human–robot relationships (MacDorman and Cowley, 2006; Miklósi et al., 2017). Moreover, as both children and parents are stakeholders, their individual differences and interaction patterns could predict the success of storytelling robots. One critical variable is parenting style, which correlates with children’s technology use (Chou and Fen, 2014). In addition, to evaluate parental acceptance of children’s storytelling robots more accurately and to explore how the robots would be brought into the family, longitudinal studies are needed. Finally, more generalizable studies to support the proposal of the uncanny valley of AI could come from future work, including surveys, replications with a broader sample, and laboratory experiments.

## Conclusion

The present exploratory study investigated parental acceptance of storytelling robots for young children in the home, a subject that has received scant attention. Using design fiction as a research technique, we found that household storytelling robots are more than a new type of technology for children. They provide an intricate testing ground for studies on cognitive perception, family dynamics, and human–robot interaction design.

Our findings showed that parents had ambivalent though generally positive attitudes toward storytelling robots and were willing to accept them in the home. Parents valued storytelling for their child’s literacy education, habit cultivation, and family bonding. These goals provide a framework for assessing the usefulness of storytelling robots. Likely predictors of robot acceptance include context of use, perceived agency, and perceived intelligence. Parents both valued and felt concern about the robot’s adaptive and affective capability.

We discussed possible mental models and cognitive mechanisms behind parental expectations. Unlike screen-based technologies, parents could see a storytelling robot as a parent double, which could relieve them of boring and stressful aspects of parenting but could also threaten parenthood. We also introduced the concept of an uncanny valley of AI to explain some of the parents’ ambivalent views. Parents found it difficult to establish a mental model of how a robot with a social capability operates, which creates cognitive dissonance and a feeling of uncanniness. This feeling might be mitigated by making its AI more transparent and understandable. Finally, we explored the implications of using robots for children’s story time, including their potential influence on parental well-being, and suggested directions for future research.

## Data Availability

The raw data supporting the conclusions of this article will be made available by the authors without undue reservation.

## Footnotes

1 In the U.S., 83% of primary caregivers are mothers (Laughlin, 2013).

2 Manufacturer’s website for Luka, https://luka.ling.ai/, and Trobo, https://mytrobo.com/.
