# Supplementary material for: Parental Acceptance of Children’s Storytelling Robots: A Projection of the Uncanny Valley of AI
Source: Front Robot AI. 2021 May 19;8:579993. doi: 10.3389/frobt.2021.579993 (PMC8172185; doi:10.3389/frobt.2021.579993)
Supplement: Supplementary file 1 [file datasheet1.docx]

**Outline of Semi-structured Interview Questions**

1. Do you tell your child stories?
   1. When, where, why, and how?
   2. What do you enjoy or not enjoy about it?
2. What do you think about having a children’s storytelling robot?
   1. Are there any advantages or disadvantages of using a storytelling robot? Why?
   2. Would you like your child to play alone with the robot or not? When and why?
   3. Would you consider having a storytelling robot for your child? Why or why not?
3. How would you envision a child’s storytelling robot?
   1. What factors would affect your decision in choosing a storytelling robot?
   2. What kind of interaction would you expect a robot to perform in a storytelling activity?
   3. What physical appearance would you desire for a robot?
   4. What kinds of stories would you expect a robot to tell your child?
   5. What level of intelligence would you expect a storytelling robot to have?
   6. What do you think is the role of a robot in story time?
   7. How do you think the modality of a robot might be different from other technology you used in telling your child stories?
   8. Do you have any other comment, suggestions, or concerns related to storytelling robots for children?
4. What is your experience and general opinion about robots?
   1. Do you have any experience with robots?
   2. Do you have any robots at home? If so, how has your experience been?
   3. What jobs do you think a robot could help you with in your home?
